# Comparative transcriptome analysis identified important genes and regulatory pathways for flower color variation in *Paphiopedilum hirsutissimum*

**DOI:** 10.1186/s12870-021-03256-3

**Published:** 2021-10-27

**Authors:** Xiuling Li, Jizheng Fan, Shuming Luo, Ling Yin, Hongying Liao, Xueqiang Cui, Jingzhou He, Yanhua Zeng, Junjie Qu, Zhaoyang Bu

**Affiliations:** 1 Flower Research Institute, Guangxi Academy of Agricultural Sciences, Nanning, 530007 Guangxi China; 2 Guangxi Key Crop Genetic Improvement and Biotechnology Laboratory, Guangxi Academy of Agricultural Sciences, Nanning, 530007 Guangxi China

**Keywords:** Paphiopedilum hirsutissimum, Albino, Anthocyanin, Carotenoids, Flower color, Comparative transcriptome

## Abstract

**Background:**

*Paphiopedilum hirsutissimum* is a member of Orchidaceae family that is famous for its ornamental value around the globe, it is vulnerable due to over-exploitation and was listed in Appendix I of the Convention on International Trade in Endangered Species of Wild Fauna and Flora, which prevents its trade across borders. Variation in flower color that gives rise to different flower patterns is a major trait contributing to its high ornamental value. However, the molecular mechanism underlying color formation in *P. hirsutissimum* still remains unexplored. In the present study, we exploited natural variation in petal and labellum color of Paphiopedilum plants and used comparative transcriptome analysis as well as pigment measurements to explore the important genes, metabolites and regulatory pathways linked to flower color variation in *P. hirsutissimum*.

**Result:**

We observed that reduced anthocyanin and flavonoid contents along with slightly higher carotenoids are responsible for albino flower phenotype. Comparative transcriptome analysis identified 3287 differentially expressed genes (DEGs) among normal and albino labellum, and 3634 DEGs between normal and albino petals. Two genes encoding for flavanone 3-hydroxylase (F3H) and one gene encoding for chalcone synthase (CHS) were strongly downregulated in albino labellum and petals compared to normal flowers. As both F3H and CHS catalyze essentially important steps in anthocyanin biosynthesis pathway, downregulation of these genes is probably leading to albino flower phenotype via down-accumulation of anthocyanins. However, we observed the downregulation of major carotenoid biosynthesis genes including VDE, NCED and ABA2 which was inconsistent with the increased carotenoid accumulation in albino flowers, suggesting that carotenoid accumulation was probably controlled at post-transcriptional or translational level. In addition, we identified several key transcription factors (MYB73, MYB61, bHLH14, bHLH106, MADS-SOC1, AP2/ERF1, ERF26 and ERF87) that may regulate structural genes involved in flower color formation in *P. hirsutissimum.* Importantly, over-expression of some of these candidate TFs increased anthocyanin accumulation in tobacco leaves which provided important evidence for the role of these TFs in flower color formation probably via regulating key structural genes of the anthocyanin pathway.

**Conclusion:**

The genes identified here could be potential targets for breeding *P. hirsutissimum* with different flower color patterns by manipulating the anthocyanin and carotenoid biosynthesis pathways.

**Supplementary Information:**

The online version contains supplementary material available at 10.1186/s12870-021-03256-3.

## Background

*Paphiopedilum hirsutissimum* is one of the important members of Orchidaceae family having 736 genera. It is the second largest family of flowering plants mainly famous for its aesthetic and ornamental values [8]. Genus Paphiopedilum is known by common name Venus or lady’s slipper due the resemblance of their labellum or synsepalum with shoe. The 75 species of this genus are distributed worldwide [33]. Recently, most species of *Paphiopedilum* have faced a rapid decline, becoming endangered due to its narrow distribution, climate change, habitat loss, and overcollection for their beautiful, unique flowers. They were listed in Appendix I of the Convention on International Trade in Endangered Species of Wild Fauna and Flora, which prevents them from being traded across borders [61]. *P. hirsutissimum* is mainly prevalent in Southern China and India, and is well adapted to cultivation. Flower color in orchids is very fascinating trait, essential for the ornamental value [7]. Floral color variations mainly depend upon pigment formation and the mechanism is well characterized in many plant species [48].

Important pigments which depict floral colors are anthocyanins, carotenoids and betalains, however, betalains are found only in some species [20]. Anthocyanins are water soluble phenolic compounds providing different colors to flowers, fruits, leaves, stems and seeds [48, 67]. Anthocyanins not only give blue, red, white, orange, purple and pink colors to flowers and fruits [55] but also responsible for red-purple colors in leaves during senescence [32]. Anthocyanin biosynthesis is a complex multistep process involving many structural and regulatory genes [71]. These structural genes code enzymes like chalcone synthase (CHS), chalcone isomerase (CHI), flavanone 3-hydroxylase (F3H), dihydroflavonol 4-reductase (DFR), anthocyanidin synthase (ANS), and UDP-glucose:flavonoid 3-o-glucosyltransferase (UFGT), flavonoid 3′,5′-hydroxylase (F3’5’H), flavonoid 3′ –hydroxylase (F3’H), glutathione S transferase (GST), cinnamate 4-hydroxylase (C4H), 4-coumaroyl CoA ligase (4CL), leucoanthocyanidin dioxygenase (LDOX), phenylalanine ammonia-lyase (PAL) [15, 20, 24]. Stable form of anthocyanins having basic structure of flavylium ion, are derived from three basic anthocyanidins and many glycosides [73]. Various structural genes controlling anthocyanin biosynthesis are well characterized in many plant species [21, 27, 34]. However, studies regarding the identification of such genes in *P. hirsutissimum* are rare. Thus, identification of new genes involved in anthocyanin biosynthesis pathway in *P. hirsutissimum* will significantly improve our knowledge in the regulation of anthocyanin biosynthesis and flower color variation.

Another important non-chlorophyll pigments that give yellow to red color to plant organs are carotenoids [9]. Apart from their major role in color formation, carotenoids are also an important determinant of nutritional value of fruits and vegetables [9]. The key enzymes involved in multistep metabolic pathway of carotenoid biosynthesis are well characterized in many plant species. Most important structural genes of this pathway codes enzymes including phytoene synthase (PSY), phytoene desaturase (PDS), ζ-carotene desaturase (ZDS), carotene isomerase (CRTISO), lycopene β-cyclase (β-LCY), lycopene ε -cyclase (ε-LCY), zeaxanthin epoxidase (ZEP) and Hyb [4]. Several studies have elucidated the genes encoding key enzymes of the carotenoid biosynthesis pathway [12, 53]. Transcriptional regulation of these genes was shown to be involved in carotenoid accumulation in plants [12]. In Oncidium orchids, carotenoids were shown to be involved in yellow color of their lip [10]. However, no detailed study has been conducted to elucidate the genes and molecular mechanism of carotenoid biosynthesis with respect to color formation in *P. hirsutissimum*.

Besides the structural genes, many transcription factors (TFs) also have prominent roles in determining flower colors by affecting anthocyanin and carotenoid biosynthesis pathways [25, 30]. These TFs most often regulate the expression of important structural genes and are referred to as regulatory genes [1, 16, 70]. These TFs belong mainly to R2R3-MYB, basic helix loop helix (bHLH), MADS box and ethylene response elements (ERF) families [5, 14, 23, 28, 54]. The MYB and bHLH TFs act by forming a MYB-bHLH-WD40 protein complex to bind with the promoters of anthocyanin biosynthesis genes to regulate their expression [47, 65]. The complex formation between TTG1 (WD40), GL3/EGL3/TT8 (bHLH), and MYB75/MYB90/MYB113/MYB114 (MYB) to control anthocyanin biosynthesis was demonstrated in Arabidopsis [17, 56]. Many MYB TFs were also identified in Orchidacea family such as *DhMYB2* in *Dendrobium spp*. [35, 64], *OgMYB1* in *Oncidium spp*. [11], *PeMYB2*, *PeMYB11*, *PeMYB12* in *Phalaenopsis* spp. [25]. Any change in the expression of these particular TFs could affect the pattern of flower color [28, 54]. Thus, identification of new genes encoding these TFs in *P. hirsutissimum* will help to understand the molecular basis of flower color variation in orchids.

Transcriptome profiling has contributed significantly in identification of new genes for many important traits [63, 69]. It often provides a basis for functional characterization of new genes involved in a variety of functions such as development and stress-responsiveness [63, 69]. In this study, we used RNA-seq based transcriptome analysis to identify important genes and TFs involved in flower color variation in *P. hirsutissimum*. We have identified several structural genes and important TFs regulating key enzymes of anthocyanin and carotenoid biosynthesis pathways. Our study provides a basis for functional characterization of several candidate genes for flower color variation and to understand its mechanism in orchids. The knowledge would be helpful in breeding *P. hirsutissimum* genotypes with a variety of flower color patterns.

## Methods

### Plant materials and growth conditions

Two variants of *P. hirsutissimum* are used in the present study. The plant materials are available at the *Paphiopedilum* Ex Situ Conservation Germplasm Resource Nurseries (22°78′N, 108°27′E) of the Flower Research Institute, Guangxi Academy of Agricultural Sciences, Nanning, China. The color of the petals and labellum were confirmed by the RHS Color Chart. Normal flowers have red-purple petals from tip to center with grey-brown at the base (NP) and grey-brown labellum with brown spots (NL), while its albino variant has light yellow green petals from tip to center with yellow-green part at the base (AP) and yellowish green labellum (AL). Paphiopedilum plants were grown in a greenhouse under natural light at 30/25 °C (day/night) and watered regularly as needed. Floral tissues from above mentioned flower types (NP, NL, AP and AL) were collected in the daytime to avoid any effect on gene expression due to differences in circadian rhythms. The light yellow green part from the tip to the center of the petals of the albino variant, the red-purple part from the top to the center of the petals of the normal species, and all the labellum petals of the normal species and albino species were taken from individual plants and completed three biological replicates. Samples were kept in aluminum foil and put immediately in liquid nitrogen and stored at − 80 °C freezer until use.

### Biochemical analysis

Flavonoids, anthocyanins and carotenoids content in the petals and labellums were identified and quantified by using an LC-ESI-MS/MS system [62].

### RNA isolation and cDNA library preparation

Total RNA was isolated from collected tissues using RNAprep Pure Plant Kit (Beijing, China) following manufacturer’s protocol [69]. RNA integrity and concentration were determined on agarose gel and Nanodrop, respectively. High quality messenger RNA was treated with Oligo (dT) and subjected to fragmentation to create short fragments. After purification of cDNA, polyA tails were added to both ends, ligated with adapters and used for library preparation. A total of 12 cDNA libraries were sequenced using Illumina HiSeq™ 2000 to obtain short sequences from both ends.

### De novo transcriptome assembly and annotation

Raw reads were filtered to get clean reads by removing sequences with adapters, and low-quality transcripts. Due to absence of reference genomes, the clean reads were de novo assembled into transcripts using Trinity (version: v2.9.0) [19]. This assembly has a high degree of completeness with Benchmarking Universal Single-Copy Orthologs (BUSCO) score of 88%, which includes 1267 complete BUSCOs (88%), 1112 complete single-copy BUSCOs (77.2%), 155 complete duplicated BUSCOs (10.8%), 42 fragmented BUSCOs (2.9%), and 131 missing BUSCOs (9.1%), out of 1440 total BUSCO groups searched. Further details of the number of transcripts in each group are explained in results section. The functions of the unigenes were annotated from NCBI non-redundant protein (NR), Swiss-Prot, Clusters of orthologous groups for eukaryotic complete genomes (KOG), Gene Ontology (GO) and Kyoto Encyclopedia of Genes and Genomes (KEGG) databases using diamond software, and mapped to Pfam databases by HMMER.

### Analysis of differentially expressed genes

Gene expression levels were calculated and normalized using mapped reads by the fragments per kilobase of transcript per million mapped reads (FPKM) method using bowtie [58]. DESeq2 software was used to compare the differential expression levels between two samples [6]. Differentially expressed genes (DEG) were identified based on |log2FC| > 1 and the false discovery rate (FDR) < 0.05. GO and KEGG pathway enrichment analysis were performed from significant DEGs using R software.

### PCA, correlation and transcription factor analysis

Principal component analysis (PCA) was performed using FPKM values to identify the sample clusters and distribution pattern. Correlation analysis was performed using FPKM values to check the association of data among the replicates and comparing with other samples. PCA and correlation were performed using R studio. Venn diagram and heatmaps were generated using TBtools software.

To identify the DEGs that belong to transcription factor families, unigenes sequences were aligned using BLASTx to the plant transcription factor database (http://planttfdb.cbi.pku.edu.cn/index.php) that includes 58 plant transcription factor families from 165 plant species. Candidates that contained DNA binding domains were recognized by GO annotation for the final TF identification.

### Quantitative real time PCR analysis

Around 5 μg of total RNA was reverse transcribed into complementary DNA (cDNA) using HiScript II Q RT Supermix (Vazyme, China). For qPCR reaction, ChamQ SYBR qPCR Master Mix was used in an ABI Prism 7500 sequence detection system [68]. Transcript level normalization was done using the endogenous Actin gene. The relative gene expression was calculated using the 2^–ΔΔCT^ method described by [39]. Primers were designed using PrimerQuest Tool (http://sg.idtdna.com/Primerquest/Home/Index) and the sequences are presented in supplementary Table 1.

### Vector construction and genetic transformation in tobacco

Target genes were amplified from *P. hirsutissimum* cDNA samples using homologous recombination primers with 20-bp 5′ upstream sequence including 14-bp vector homologous sequence followed by 6-bp of restriction enzyme site. The primer sequences are mentioned in supplementary Table S1. The empty vectors of pCAMBIA2300-GFP plasmid were linearized using double enzyme digestion *SacI* and *XbaI.* The amplified fragments of target genes were cloned into linearized vectors of pCAMBIA2300-GFP under CamV35s promoter using NovoRec® plus One step PCR Cloning Kit (Novoprotein, China). The positive transformants of *E. coli* were confirmed by PCR followed by sequencing. The recombinant plasmids containing target gene were then transformed into *Agrobacterium tumefacien* using electroporation method and single colonies were selected on LB plates with kanamycin antibiotic. The *A. tumefacien* containing recombinant plasmids were transformed into tobacco leaves using needle-less syringe and kept in dark at room temperature until further analysis.

### Statistical analysis

Data was analyzed using R language. All assays were performed in triplicate. Mean values were compared by analysis of variance (ANOVA) combined with Duncan’s multiple range tests.

Normal flowers have red-purple petals from tip to center with grey-brown at the base (NP) and grey-brown labellum with brown spots (NL), while its albino variant has light yellow green petals from tip to center with yellow-green part at the base (AP) and yellowish green labellum (AL).

## Results

### Phenotypic and biochemical characterization of *P. hirsutissimum* flowers

**Fig. 1:**
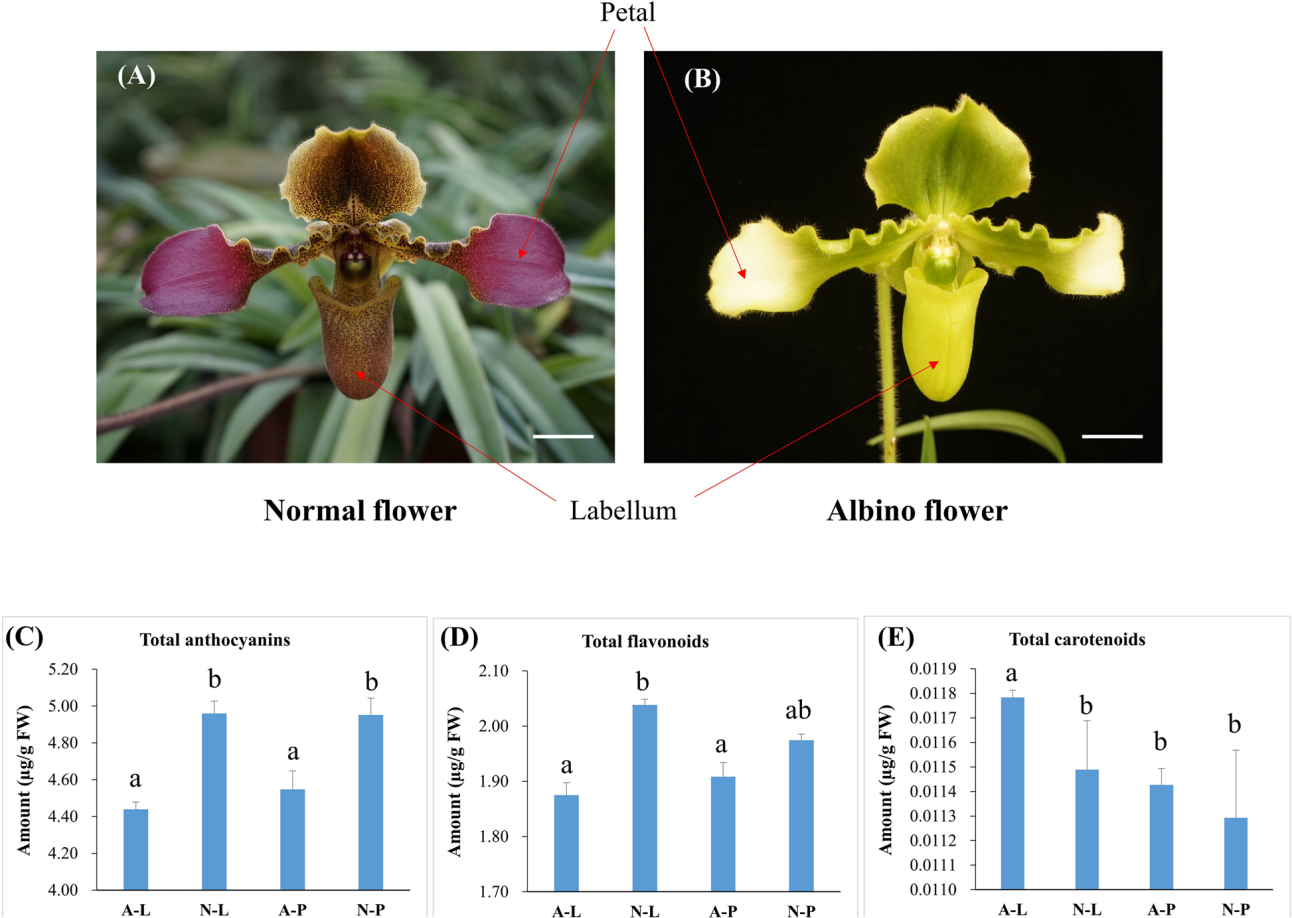
Phenotypic comparison and biochemical profile of normal and albino flower of *Paphiopedilum hirsutissimum.* **A** and **B** Labellum and petals of normal flower (**A**) and albino variant (**B**). Scale bars = 25 mm. **C**, **D** and **E** Determination of total anthocyanins (**C**), total flavonoids (**D**) and total carotenoids (**E**) from labellum and petals of normal and albino flowers. Different letters above bars represent significant difference at *P* < 0.05. Data represent the means of three biological replicates ± SD

Two flower types of *P. hirsutissimum* were selected in the present study. Normal flower shows red-purple petals from tip to center with grey-brown at the base (NP) and grey-brown labellum with brown spots (NL) but its albino variant shows light yellow green petals from tip to center with yellow-green part at the base (AP) and yellowish-green labellum without purple spots (AL) (Fig. 1a,b). To understand the biochemical basis of flower color variation in *P. hirsutissimum*, we measured anthocyanins, carotenoids and flavonoid contents. The biochemical profile showed a considerable decrease in total anthocyanins in AL and AP compared to NL and NP, respectively (Fig. 1c). Similarly, total flavonoids were also reduced in AL and AP compared to NL and NP, respectively (Fig. 1d). In contrast, we observed a slight increase in total carotenoids in AL and AP compared to NL and NP, respectively (Fig. 1e). These findings suggest a possible positive correlation of anthocyanins and flavonoids with normal flower phenotype, and that of carotenoids with albino phenotype. In other words, reduced anthocyanin and flavonoid contents along with slightly higher carotenoids are responsible for albino flower phenotype (AL and AP).

### Analysis of transcriptome dataset

**Fig. 2:**
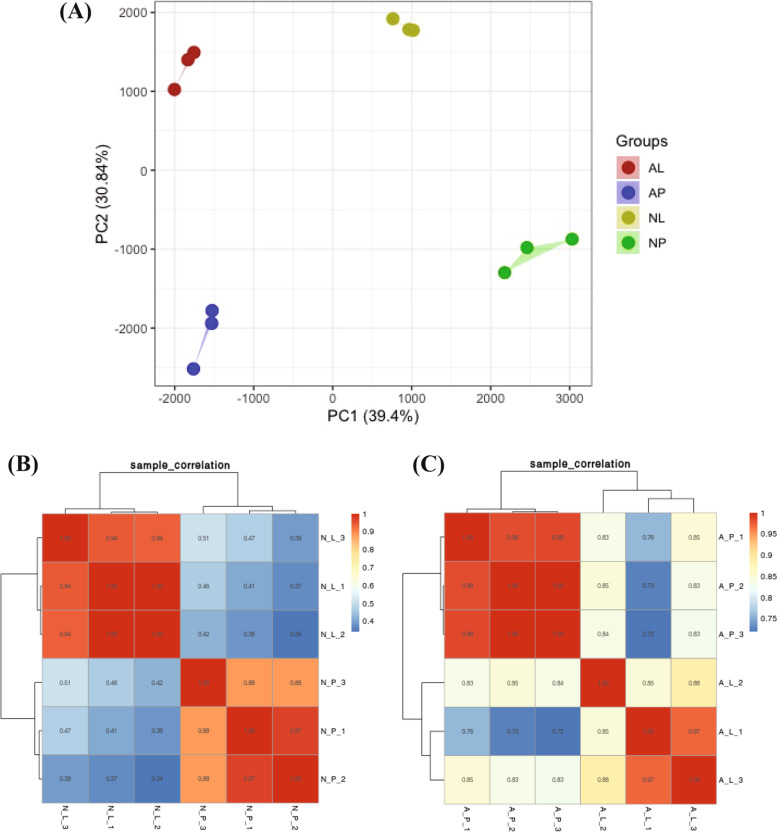
Principal component and correlation analysis of labellum and petal tissues from normal and albino flowers in *Paphiopedilum hirsutissimum*. **A** Principal component analysis based on FPKM values from 3 replicates of each tissue. NL, normal labellum; NP, normal petal; AL, albino labellum; AP, albino petal. **B** Pearson correlation analysis between replicates of normal flower labellum and petal. **C** Correlation analysis between replicates of albino flower labellum and petal. N_L, normal labellum; N_P, normal petal; A_L, albino labellum; A_P, albino petal. The number 1, 2 and 3 with each sample represents number of replicates

To understand the molecular basis of flower color variation and differential anthocyanin and carotenoid contents, transcriptome analysis of normal (NL and NP) and albino (AL and AP) flower tissues was performed. Using 3 biological replicates for each sample, a total of 12 cDNA libraries were developed and used for transcriptome analysis (RNA-seq). A total of 18,236,750–21,697,775 reads were obtained which were de novo assembled into transcripts. Transcript assembly led to the identification of 34,806 transcripts in normal samples (NL and NP) with an N50 score of 1194 kb. For albino samples (AL and AP), 28,805 transcripts were obtained with the N50 score of 1341 kb. Benchmarking Universal Single-Copy Orthologs (BUSCO) program revealed that the integrity of transcripts of normal samples (NL and NP) was 83.1% with redundancy rate of 6.6%, while integrity for albino samples (AL and AP) was 84.7% with 8.9% redundancy rate. This shows a high quality of transcriptome dataset to study further expression profiles of samples. The gene expression levels of unigenes were determined in term of fragments per kilobase of exon per million fragments mapped (FPKM) values. Principal component analysis (PCA) based on FPKM values divided the samples in 4 distinct groups with each sample making a separate group with its replicates (Fig. 2a). Samples from normal flower (NP and NL) fell on the positive side of X-axis while samples from albino flower (AP and AL) fell on the negative side of X-axis. PCA analysis further showed that major portion of total variation (70.24%) consists of two principal components (PC1: 39.4%, PC2: 30.84%). Correlation analysis indicated a significant positive correlation among the 3 replicates of each sample, while a low level of correlation was observed with other samples (Fig. 2b,c). This suggests a consistent gene expression levels among all the biological replicates and proves the reliability of expression analysis.

### Validation of RNA-seq dataset using qRT-PCR

**Fig. 3:**
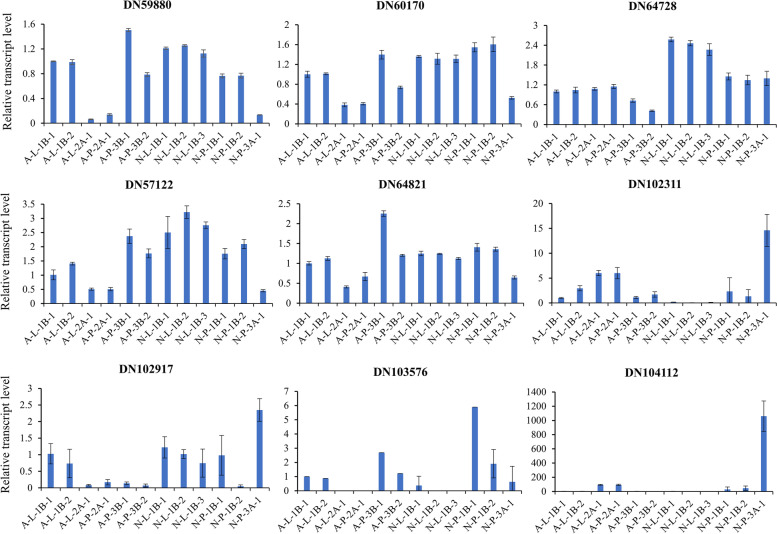
qRT-PCR analysis for validation of RNA seq data. Data is mean of 3 biological replicates with ± SD

Before proceeding for further analysis of transcriptome data, we attempted to validate the expression trend using qRT-PCR analysis. To do this, we have selected 9 DEGs from transcriptome dataset showing variable expression and performed qRT-PCR analysis (Fig. 3). According to qRT-PCR analysis, all genes have shown an expression trend similar to the transcriptome dataset, which indicates that the quality of our transcriptome data is good enough to produce reproducible results and is worthy to proceed for further downstream analysis.

### Comparison of DEGs among the normal and albino tissues

**Fig. 4:**
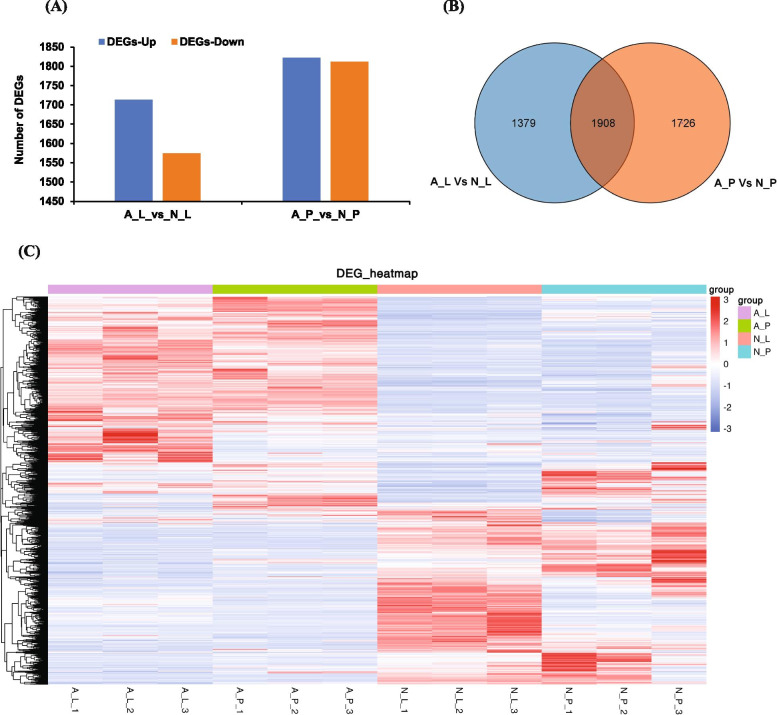
Analysis of differentially expressed genes (DEGs) between labellum and petals of normal and albino flower in *Paphiopedilum hirsutissimum*. **A** Number of up- and down-regulated genes between normal and albino tissues. **B** Venn diagram showing the shared and specific number of DEGs between labellum and petal tissues. **C** Heatmap showing expression pattern of DEGs in each sample based on FPKM values. N_L, normal labellum; N_P, normal petal; A_L, albino labellum; A_P, albino petal. The number 1, 2 and 3 with each sample represents number of replicates

To identify the differentially expressed genes (DEGs) between the normal and albino samples, we performed a tissue-wise comparison of DEGs by dividing into two groups; AL vs NL (labellum tissue) and AP vs NP (petal tissue). Comparative analysis identified 3287 DEGs (1713 up-regulated and 1574 down-regulated genes) between AL vs NL (Table S2, Fig. 4a), and 3634 DEGs (1822 up-regulated and 1812 down-regulated genes) between AP vs NP (Table S3, Fig. 4a). Next, we identified the shared and specific number of DEGs between labellum and petal tissues. Venn diagram indicated that 1379 genes were expressed only in AL vs NL; 1726 genes were expressed only in AP vs NP; while 1908 DEGs were common between both groups (Fig. 4b). Heatmaps along with hierarchical clustering showed distinct patterns of gene expression among the normal and albino tissues (Fig. 4c). The expression pattern between the AL and AP were more similar compared to NL and NP suggesting a distinct pattern of gene expression among the normal and albino flowers. To look further into details, we attempted to identify the DEGs expressed only in normal tissues of paphiopedilum. Importantly, 39 DEGs were uniquely expressed in the petals and labellum of normal flowers with no expression in albino tissues (Supplementary Fig. S1a). Similarly, 4 DEGs were solely expressed in the normal labellum tissues and not expressed in any other tissue (Supplementary Fig. S1b). This suggests the key role of these 39 and 4 DEGs in color formation in petal and labellum tissues.

### Gene ontology and KEGG enrichment

We performed GO enrichment analysis and revealed the significantly enriched biological pathways between AL vs NL and AP vs NP (Supplementary Fig. S2). The most enriched GO terms between AL vs NL were photosynthesis, response to auxin, response to oxidative stress, and cell wall modification. The most enriched GO terms between AP vs NP were DNA metabolic process and regulation of gene expression. Since albino flower has more green color than normal flower, it may have more prolonged photosynthesis as shown in GO enriched term. The KEGG enrichment analysis for AL vs NL showed the consist results as GO enrichment with photosynthesis, plant-hormone signal transduction and phenylpropanoid as most enriched biological pathways (Supplementary Fig. S3a). Plant-hormone signal transduction, plant-pathogen interaction and phenylpropanoid biosynthesis were revealed as most enriched pathways between AP vs NP (Supplementary Fig. S3b). These findings suggest the involvement of photosynthetic pigments and phytohormones in flower color variation in Paphiopedilum.

### DEGs involved in anthocyanin biosynthesis pathway in Paphiopedilum flowers

**Fig. 5:**
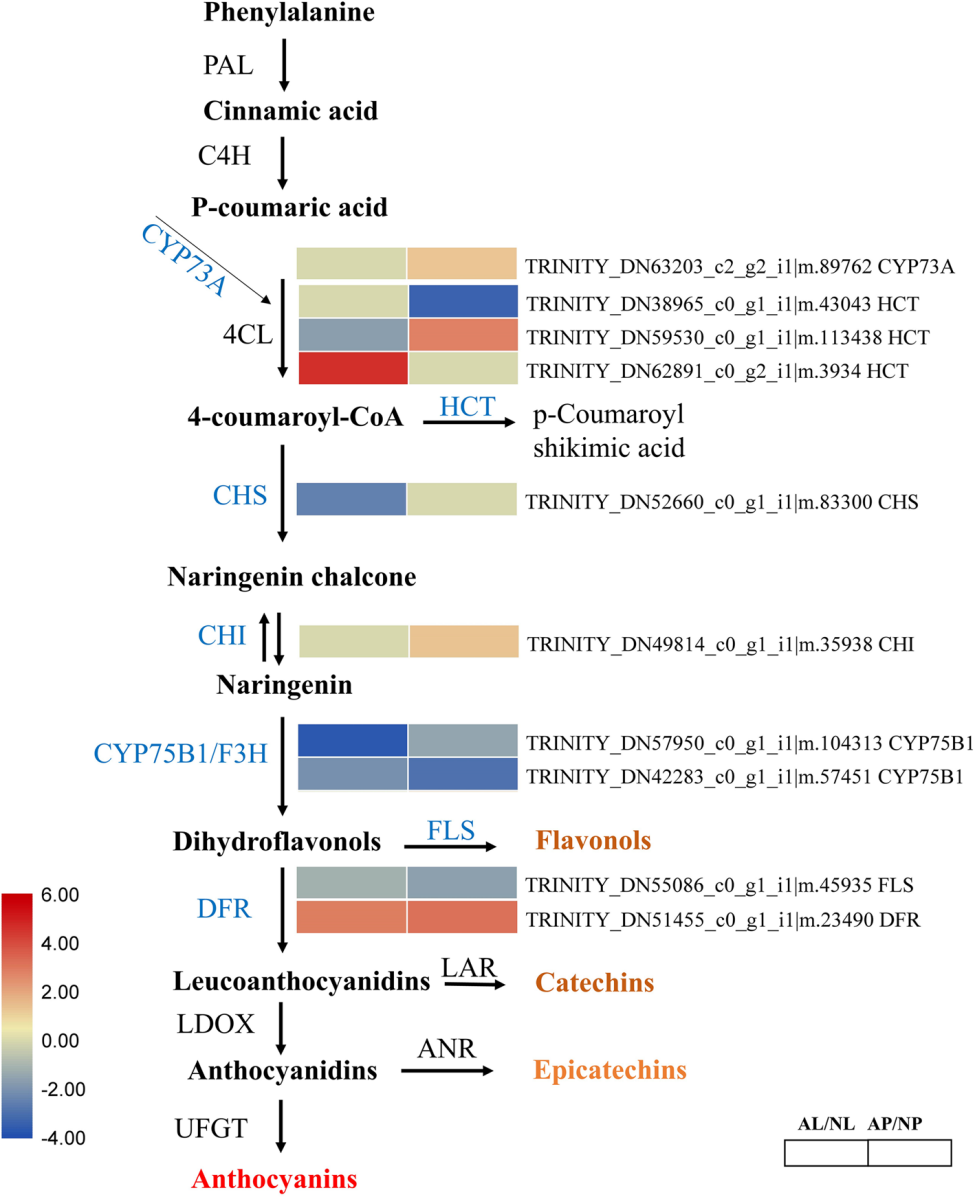
Overview of anthocyanin biosynthesis pathway and involvement of differentially expressed genes (DEGs) in *Paphiopedilum hirsutissimum* flower. Heatmaps were generated using Log2FC based on FPKM values and the DEGs encoding specific enzymes are denoted in blue color. In scale bar, red color shows upregulation, white color shows downregulation, and orange color means no change. Phenylalanine ammonia lyase (PAL), 4-coumarateCoA ligase (4CL), chalcone synthase (CHS), chalcone isomerase (CHI), flavone 3-hydroxylase (F3H), flavonol synthase (FLS), dihydroflavonol reductase (DFR), and UDP-flavonoid glucosyl transferase (UFGT), and leucoanthocyanidin dioxygenase (LDOX). NL, normal labellum; NP, normal petal; AL, albino labellum; AP, albino petal

To further explore the mechanism of flower color formation in *P. hirsutissimum*, a more detailed analysis of genes involved in color related pathways is required. To study the differential regulation of anthocyanin biosynthesis in *P. hirsutissimum* flowers, we mapped the DEGs related to anthocyanin biosynthesis pathway. Ten genes were found to be differentially expressed between AL vs NL and AP vs NP at different steps of anthocyanin biosynthesis pathway (Fig. 5). Shikimate O-hydroxycinnamoyl transferase (HCT) is an enzyme [EC:2.3.133] that catalyzes the formation of p-Coumaroyl shikimic acid (a by-product of anthocyanin pathway) from 4-coumaroyl-CoA. Three genes encoding HCT (*TRINITY_DN38965_c0_g1_i1|m.43043*, *TRINITY_DN59530_c0_g1_i1|m.113438* and *TRINITY_DN62891_c0_g2_i1|m.3934*) were differentially expressed between AL vs NL and AP vs NP (Fig. 5). Chalcone synthase (CHS) is a key enzyme [EC:2.3.1.74] that catalyzes the formation of Naringenin chalcone from 4-coumaroyl-CoA. Gene encoding CHS (*TRINITY_DN52660_c0_g1_i1|m.83300*) was downregulated in AL vs NL (Log2FC: − 2.48), while no considerable change was observed in AP vs NP, suggesting the possible role of this gene in regulating labellum color formation. Chalcone isomerase (CHI) is another enzyme in the anthocyanin biosynthesis pathway that converts Naringenin chalcone to Naringenin. Gene encoding CHI (*TRINITY_DN49814_c0_g1_i1|m.35938*) was upregulated in AP compared to NP (Log2FC: 1.34), while no change was observed in labellum tissues, suggesting a role in petal color formation. Flavonoid 3′-monooxygenase (F3H/CYP75B1) catalyzes the formation of Dihydroflavonols from Naringenin and 2 genes encoding F3H were downregulated in the AL and AP tissues compared to NL and NP, respectively. It is noteworthy that both genes encoding F3H (*TRINITY_DN57950_c0_g1_i1|m.104313* and *TRINITY_DN42283_c0_g1_i1|m.57451*) were downregulated in albino tissues, which suggest that this may block color formation via reduced anthocyanin biosynthesis. Flavonols are by-product of anthocyanin pathway that is catalyzed by flavonol synthase (FLS). We observed downregulation of gene (*TRINITY_DN55086_c0_g1_i1|m.45935*) encoding FLS in both albino tissues (AL and AP) compared to normal tissues. DFR (DFR; bifunctional dihydroflavonol 4-reductase) is an important enzyme [[EC:1.1.1.219] that catalyzes the formation of leucoanthocyanidins from dihydroflavonols in anthocyanin biosynthesis [36]. A significant upregulation of DFR encoding gene (*TRINITY_DN51455_c0_g1_i1|m.23490)* was observed in both labellum and petal tissues of albino flower (Fig. 5). Together, these results identified important genes encoding key enzymes involved in anthocyanin biosynthesis which are possibly playing role in determining flower color variation in *P. hirsutissimum*.

### DEGs involved in carotenoid biosynthesis pathway in Paphiopedilum flowers

**Fig. 6:**
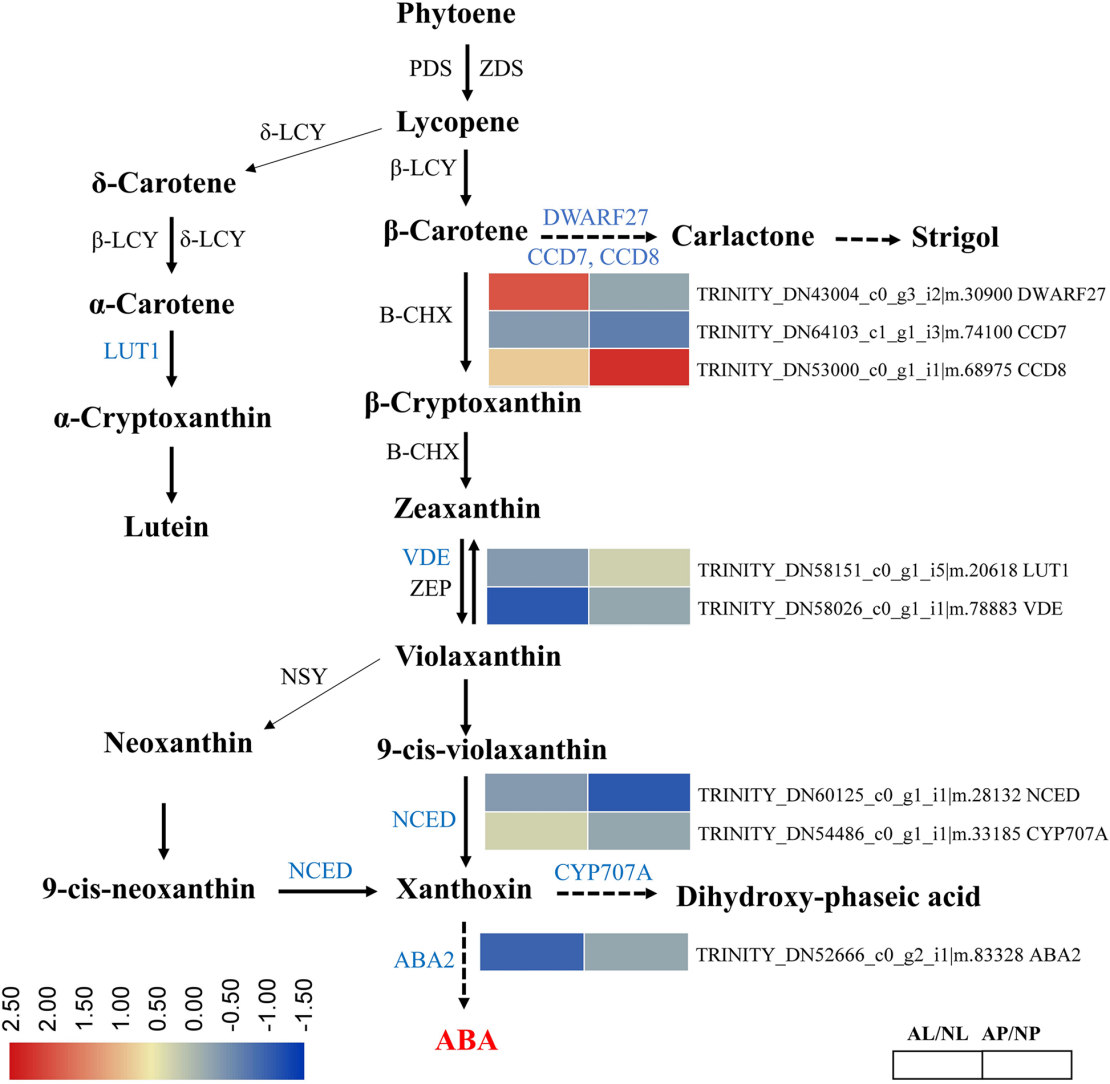
Overview of carotenoids biosynthesis pathway and involvement of differentially expressed genes (DEGs) in *Paphiopedilum hirsutissimum* flower. Heatmaps were generated using Log2FC based on FPKM values and the DEGs encoding specific enzymes are denoted in blue color. In scale bar, red color shows upregulation, white color shows downregulation, and orange color means no change. Phytoene desaturase (PDS), ζ-carotene desaturase (ZDS), lycopene β-cyclase (β-LCY), lycopene ε -cyclase (ε-LCY), β-carotene isomerase (DWARF27), 9-cis-β-carotene 9′,10′-cleaving dioxygenase (CCD7) and carlactone synthase (CCD8), zeaxanthin epoxidase (ZEP), epsilon hydroxylase (LUT1), violaxanthin de-epoxidase (VDE), 9-cis-epoxycarotenoid dioxygenase (NCED), (+)-abscisic acid 8′-hydroxylase (CYP707A), xanthoxin dehydrogenase (ABA2)

Carotenoids are important pigments in photosynthetic and non-photosynthetic organs of plants [40]. To identify the DEGs involved in flower color variation in *P. hirsutissimum* via carotenoids, we mapped DEGs in carotenoid biosynthesis pathway of KEGG. A total of 8 DEGs were identified that encode enzymes of carotenoid biosynthesis pathway (Fig. 6). β-carotene isomerase (DWARF27), 9-cis-β-carotene 9′,10′-cleaving dioxygenase (CCD7) and carlactone synthase (CCD8) are important enzymes that catalyzes the formation of carlactone, a precursor molecule of strigolactone and a by-product of carotenoid biosynthesis pathway [44]. Genes encoding DWARF27 (*TRINITY_DN43004_c0_g3_i2|m.30900*) and CCD8 (*TRINITY_DN53000_c0_g1_i1|m.68975*) were upregulated while CCD7 (*TRINITY_DN64103_c1_g1_i3|m.74100*) was downregulated in the albino flower tissues compared to the normal tissues (Fig. 6). Lutein is another carotenoid molecule, and gene encoding carotenoid epsilon hydroxylase (LUT1) (*TRINITY_DN58151_c0_g1_i5|m.20618)* for lutein biosynthesis from α-carotene was upregulated in albino petals compared to normal petals, however, no change was observed in labellum tissues. Violaxanthin de-epoxidase (VDE) is another important enzyme that catalyzes the formation of violaxanthin from zeaxanthin [37]. A VDE encoding gene (*TRINITY_DN58026_c0_g1_i1|m.78883*) was significantly downregulated in the albino labellum compared to the normal sample (Fig. 6). 9-cis-epoxycarotenoid dioxygenase (NCED) is the key enzyme in the ABA synthesis and gene encoding NCED (*TRINITY_DN60125_c0_g1_i1|m.28132*) was significantly downregulated in albino petals (AP) compared to the normal sample which suggests the down-accumulation of xanthoxin (Fig. 6). In contrast, gene encoding (+)-abscisic acid 8′-hydroxylase (*CYP707A*) (*TRINITY_DN54486_c0_g1_i1|m.33185)* was upregulated in AL tissues that seems to enhance the formation of dihydroxy-phaseic acid, a by-product of carotenoid biosynthesis pathway. Xanthoxin dehydrogenase (ABA2) is another key enzyme that catalyzes the last step of ABA biosynthesis. Gene encoding ABA2 (*TRINITY_DN52666_c0_g2_i1|m.83328*) was also downregulated in AL tissues. Notably, we observed downregulation of major carotenoid pathway genes (VDE, NCED and ABA2) which was inconsistent with increased carotenoid accumulation.

### DEGs encoding the key transcription factors related to color formation

**Fig. 7:**
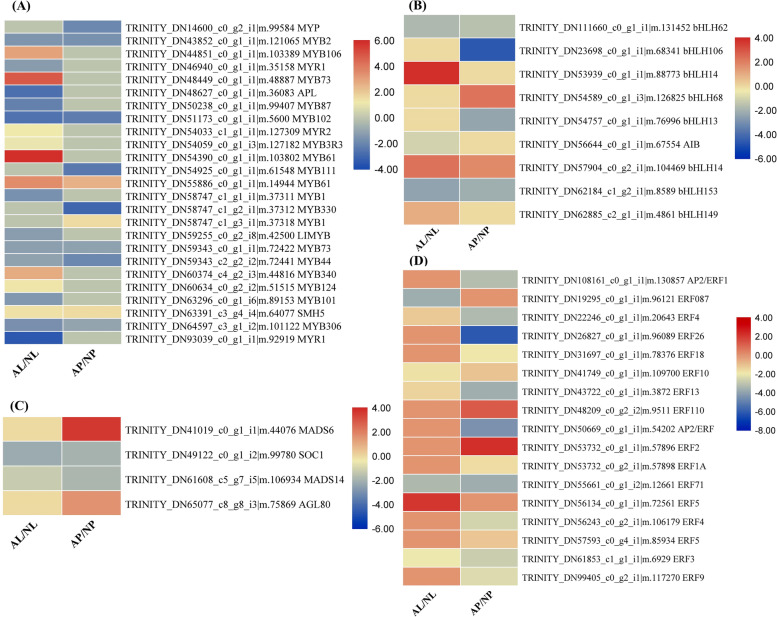
Expression profile of transcription factor families involved in flower color formation. Differentially expressed genes (DEGs) encoding MYB (**A**), bHLH (**B**), MADS-Box (**C**) and ERF (**D**) transcription factors between labellum and petals of normal and albino flowers of *Paphiopedilum hirsutissimum.* Heatmaps were generated using Log2FC based on FPKM values

Some transcription factor (TF) families including MYB, bHLH, MADS-Box and ERF play important roles in color formation via anthocyanin biosynthesis by regulating the expression of key structural genes. We therefore analyzed the expression pattern of these TF families in AL vs NL and AP vs NP tissues (Fig. 7). Twenty-five MYB TF encoding genes were differentially expressed among normal and albino flowers (Fig. 7a). Of these 25 genes, 14 were differentially expressed only in labellum tissues, 4 were specific to petal tissues, and 7 DEGs were differentially expressed in both labellum and petal tissues (Fig. 7a). In labellum tissue, 12 MYB genes were downregulated while 9 MYB genes were upregulated in albino flower. In petal tissue, 8 MYB genes were downregulated while 3 MYB genes were upregulated in albino flower. This could potentially affect the expression of structural genes related to color formation in albino mutant leading to loss of red-purple spots in albino flowers. Nine genes encoding bHLH TFs were found to be differentially expressed in albino vs normal flowers (Fig. 7b). Of these 9 genes, 3 were downregulated while 3 were upregulated in AL vs NL, while 4 bHLH genes were downregulated and 2 were upregulated in AP vs NP. Four MADS-Box genes were identified, of which 2 were downregulated in both labellum and petal tissues, while 2 were upregulated only in petal tissues (Fig. 7c). Lastly, we identified 17 DEGs encoding ERF and AP2/ERF TFs, and most of which were downregulated in AL and AP compared to NL and NP, respectively (Fig. 7d). For the ERF TFs, 6 were downregulated in AL vs NL, and 13 were downregulated in AP vs NP. However, 1 ERF gene was upregulated in AL, and 2 genes were upregulated in AP compared to NL and NP. Collectively, strong downregulation of MYB, bHLH, MADS-Box and ERF TFs may reduce the expression of target structural genes involved in flower color formation, leading to the albino mutant phenotype in *P. hirsutissimum.*

### Overexpression of transcription factors increased anthocyanin accumulation

**Fig. 8:**
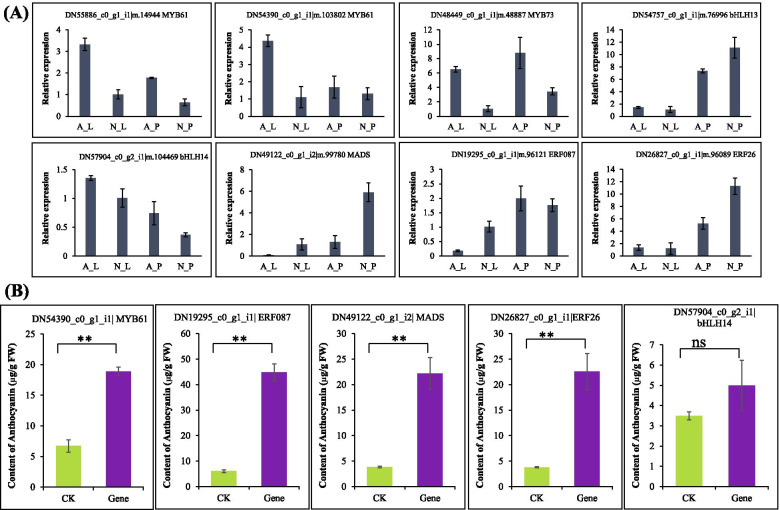
qRT-PCR and overexpression of candidate transcription factors. **A** qRT-PCR analysis of candidate transcription factors from Fig. 7 for validation of transcriptome data. Data is mean of 3 biological replicates with ± SD. **B** Anthocyanin content in tobacco leaves overexpressing candidate transcription factors. Ck = control without overexpression. Data is mean of 3 biological replicates with ± SD. ** shows significant difference at *P* < 0.01; ns = non-significant

To get a deeper insight into the role of TFs in color formation, we first validated the expression of eight TFs (3 MYB, 2 bHLH, 2 ERF and 1 MADS-Box) with contrasting expression patterns in our RNA-seq data using qRT-PCR analysis. All the TFs encoding genes showed more or less similar expression trend in our qRT-PCR data as observed in RNA-seq data (Fig. 8A). This gave us confidence to move further for the functional validation of some TFs. To see if these TFs could affect the flower color formation, we transiently overexpressed 5 TFs into tobacco (*Nicotiana benthamiana*) leaves and measured the anthocyanin accumulation, the major regulator of flower color. 4 out of 5 TFs belonging to each of the four tested TFs families significantly increased the anthocyanin accumulation in tobacco leaf compared to the control leaves (Fig. 8B). This showed the great potential of identification and utilization of potential TFs of these families in regulating flower color variation in *P. hirsutissimum*.

## Discussion

Flower industry contributes significantly to the economy of several countries such as Japan [3], Netherlands, Columbia, Ecuador (https://www.petalrepublic.com/floristry-and-floriculture-statistics/) and Kenya (http://www.kenyarep-jp.com/business/flower_e.html). *P. hirsutissimum* belongs to the family of orchids and have ornamental plants with unique flower patterns [25]. Variation in flower colors is an important trait considered for breeding new varieties of ornamental plants [3, 31]. Understanding the mechanism of color variation could help in breeding desirable genotypes and ultimately uplift the flower industry [3]. In this study, we attempted to identify the key players of flower color formation in *P. hirsutissimum* plants by using natural variation in flower color. The wild-type flowers of *P. hirsutissimum* have pink-rose petals and yellow labellum with purple spots which are very attractive to people and give a pleasant feeling. Its natural albino variant shows off-white petals with yellowish-green labellum that provides a good source of study the mechanism of flower color variation. In accordance with the albino phenotype, we observed reduced anthocyanins and increased carotenoid contents in mutant (Fig. 1). To uncover the molecular basis of flower color variation, we used the transcriptome analysis to identify important structural and regulatory genes and highlighted key pathways regulating the flower color variation in *P. hirsutissimum*.

### Critical pathways involved in regulation of flower color variation

Anthocyanin biosynthesis is a major pathway involved in flower and fruit color formation in orchids and other flowering plants [13]. The key enzymes of anthocyanin biosynthesis pathway are PAL, C4H, 4CL, CHS, CHI, F3H, F30H, FLS, DFR, ANS, and UFGT [13]. We identified 10 DEGs for anthocyanin biosynthesis among the normal and albino flower tissues encoding for the CHS, CHI, F3H, FLS and DFR (Fig. 5). Studies have shown that differential expression of genes encoding these key enzymes (CHS, CHI, F3H, FLS and DFR) play critical role in anthocyanin accumulation and affect the flower color phenotype [13, 21, 27, 72]. Importantly, we observed strong downregulation of CHS encoding gene in albino labellum. As CHS catalyzes the formation of Naringenin chalcone, an important substrate of anthocyanin pathway [66], the downregulation of CHS gene is probably leading to reduced anthocyanins by providing the low amount of substrate ultimately leading to the albino labellum phenotype [13]. The importance of CHS genes in flower pigmentation has been reported in different studies and proved by transgenic approaches [43, 51]. For example, transformation of antisense cDNA of CHS gene inhibited the flower color formation in petunia and caused white flower phenotype [59]. CHI is another important enzyme that converts Naringenin chalcone to Naringenin, a precursor molecule for several flavanols [32]. Gene encoding CHI was downregulated in the normal colored petals compared to albino petal (Fig. 5). As studies have shown the positive correlation of anthocyanin accumulation with CHI [11, 72], the downregulation of CHI encoding gene in colored petals could be due to the feedback regulation [30]. Similarly, we observed strong downregulation of two F3H(CYP75B1) encoding genes in the albino labellum and petals (Fig. 5). F3H genes have been shown to strongly associated with red color formation in flower petals and leaves in ornamental plants [26]. These F3H genes are often activated by the MYB TFs which bind to the promoters of F3H genes at conserved motifs [57]. Being the major structural gene in anthocyanin biosynthesis pathway, the downregulation of F3H encoding genes may have affected the overall anthocyanin accumulation in *P. hirsutissimum* flowers [21, 30], contributing to the loss of red-purple color in petals and labellum.

In addition to the anthocyanins, carotenoids also contribute in the color pigmentation in flowers and mainly confer yellow color [9, 40]. Here, we identified 8 DEGs encoding the key enzymes of carotenoid biosynthesis pathway (Fig. 6). Strigolactone is an important phytohormone derived from carotenoids and function in various plant developmental processes [29, 45]. Genes encoding CCD8 and DWARF27 were strongly upregulated in albino flower tissues (Fig. 6). CCD genes not only play role in strigolactone synthesis, but also play important roles in leaf senescence and flower development [52]. Thus, strong upregulation of CCD8 and DWARF27 genes in *P. hirsutissimum* flowers may play an important role in flower color variation which is not yet known. Here it is important to note that DWARF27, CCD8 and LUT1 regulate the formation of byproducts of carotenoid pathway, and upregulation of genes encoding these enzymes could be the compensatory response due to downregulation of other genes of carotenoid pathway [69]. VDE is an important enzyme that catalyzes the formation of violaxanthin [37], and we observed a significant downregulation of VDE gene in the albino labellum. NCED is another key enzyme in the ABA synthesis that catalyzes formation of xanthoxin, the precursor of ABA [22, 46]. Xanthoxin is then used as a substrate for the synthesis of ABA via an intermediate step by the action of ABA2 enzyme [18]. ABA is known to play role in diverse metabolic processes in plants from stress responses to plant development [22, 38, 74]. Its role in flower development has also been demonstrated [2]. Genes encoding these key enzymes NCED (*TRINITY_DN60125_c0_g1_i1|m.28132*) and ABA2 (*TRINITY_DN52666_c0_g2_i1|m.83328*) were downregulated in albino petals and labellum, respectively (Fig. 6). Although, the increased carotenoid level in mutants is in accordance with the albino phenotype, the reduced expression of several carotenoid pathway genes could be due to negative feedback regulation and these genes may be playing role at post-transcriptional or translational level [41]. This is quite possible because the studies have found a very weak correlation of gene expression with protein abundance for many genes [60].

### Transcriptional regulation of flower color variation

Transcription factors play a significant role in the regulation of flower color in Paphiopedilum species [25]. Among various TF families, MYB and bHLH are the most significant regulators of flower color variation. These TFs work in different ways to regulate flower color variation. They can either regulate the expression of other key genes of anthocyanin and carotenoid biosynthesis [25], or make a complex with other TFs to regulate level of anthocyanin and carotenoids [30]. Three MYB TFs *PeMYB2*, *PeMYB11*, and *PeMYB12* activate the expression of major anthocyanin biosynthetic genes *PeF3H5*, *PeDFR1*, and *PeANS3* causing the red pigmentation in flowers of *Phalaenopsis* spp. [25]. Silencing of these MYB TFs resulted in loss of red color in flowers along with reduced levels of anthocyanins in *Phalaenopsis* spp. In addition, an R2R3-MYB TF called Reduced Carotenoid Pigmentation 1 (RCP1) positively regulate the expression of carotenoid biosynthesis genes and affect carotenoid accumulation and flower color in *Mimulus lewisii* [49]. The loss-of-function mutant showed reduced carotenoid level and its overexpression restored the carotenoid production and complemented the flower phenotype [49]. Importantly, many MYB TFs contain the [D/E]Lx2[R/K]x3Lx6Lx3R motif that is predicted to interact with bHLH factors [25]. Several studies reported that MYB, bHLH and WD40 proteins make a complex and control flower color by activating the anthocyanin and carotenoid biosynthesis genes [30, 65]. Recently, an R2R3-MYB protein, WHITE PETAL1 (WP1), was shown to play a critical role in regulating floral carotenoid pigmentation in *Medicago truncatula* [42]. This WP1 TF activates the expression of carotenoid biosynthesis genes by interacting with bHLH protein MtTT8 and WDR protein MtWD40–1 [42]. In the present study, we identified 25 MYB and 9 bHLH TF encoding genes that are differentially expressed in albino and colored flowers of Paphiopedilum (Fig. 7). It is very likely that these MYB and bHLH TFs regulate the genes of anthocyanin pathway either directly or by making a complex, thereby regulating albino flower phenotype in *P. hirsutissimum*. Previously, the function of MYB genes was tested using VIGS approach by silencing some MYB genes [25]. However, with the availability of highly efficient genome editing approach, these MYB and bHLH genes could be functionally characterized using CRISPR/Cas9 mediated targeting of cis-elements or functional domains [70]. In addition to MYB and bHLH, MADS-Box and AP2/ERF TFs were also known to affect anthocyanin and carotenoid biosynthesis [5, 28]. In apple, an ERF TF *MdERF38* positively regulates anthocyanin accumulation by interacting with MdMYB1 [5]. In Bilberry, expression of a MADS box transcription factor *VmTDR4* was shown to regulate anthocyanin accumulation and fruit color by interacting with MYB TFs [28]. Importantly, some MADS-box TFs such as SIMADS1 negatively regulate carotenoid biosynthesis by downregulating the expression of carotenoid pathway genes [53]. Here, we identified 4 MADS-Box and 17 ERF or AP2/ERF TFs that were differentially expressed in *P. hirsutissimum* flowers (Fig. 7). Notably, MADS6 expression was strongly upregulated in albino petals which may be the reason of reduced expression of carotenoid biosynthesis genes. To provide another line of evidence, we overexpressed 5 TFs (at least one from each of the MYB, bHLH, ERF and MADS-Box family) in *N. benthamiana*, and 4 out of 5 TFs caused significant increase in anthocyanin accumulation (Fig. 8). This is probably due to the activation of key structural genes of anthocyanin pathway by these TFs as we observed a MYB-binding site in the promoter sequence of one of the F3H DEGs involved in catalyzing important step of anthocyanin biosynthesis pathway [21, 50]. Thus, the differential expression of TF-encoding genes is likely to be associated with flower color variation by modulating structural genes [11]. These TF encoding genes could be utilized via over-expression or silencing approaches to breed *P. hirsutissimum* plants with different flower colors [28].

## Conclusions

In this study, we used comparative transcriptome and biochemical analysis to explore the important genes and regulatory pathways linked to flower color variation in *P. hirsutissimum.* We found that downregulation of key anthocyanin biosynthesis genes F3H and CHS is probably leading to albino flower phenotype via down-accumulation of anthocyanins. Though, as per expectation, we observed an increased carotenoid accumulation in albino flowers, the expression of important carotenoid biosynthesis genes was downregulated suggesting that carotenoid accumulation was probably controlled at post-transcriptional or translational level. Lastly, we identified several key TFs (MYB73, MYB61, bHLH14, bHLH106, MADS-SOC1, AP2/ERF1, ERF26 and ERF87) that are probably regulating the expression of important structural genes. Overexpression of 4 out of 5 TFs significantly increased the anthocyanin accumulation in tobacco which provided important evidence of the role of these TFs in flower color formation. Further functional characterization of these TFs via overexpression, knock-out and protein-DNA interaction approaches could further improve our understanding of the mechanism of their action and flower color formation.

## Supplementary Information

**Additional file 1: Table S1:** Primer sequences of genes used for qRT-PCR and vector construction.

**Additional file 2: Table S2:** Differentially expressed genes (DEGs) among normal and albino labellum.

**Additional file 3: Table S3:** DEGs between normal and albino petals.

**Additional file 4: Figure S1a**: DEGs expressed only in normal flower tissues.

**Additional file 5: Figure S1b**: DEGs expressed only in normal labellum.

**Additional file 6: Figure S2**: Gene ontology enrichment analysis among normal and albino tissues.

**Additional file 7: Figure S3A**: KEGG enrichment analysis among normal and albino labellum.

**Additional file 8: Figure S3B**: KEGG enrichment analysis among normal and albino petals.

## Data Availability

The raw RNA-seq data has been submitted to NCBI SRA under the project number: PRJNA737079.

## Abbreviations

DEGs: Differentially expressed genes
TFs: Transcription factors
NL: Normal labellum
NP: Normal petal
AL: Albino labellum
AP: Albino petal
PAL: Phenylalanine ammonia lyase
4CL: 4-coumarateCoA ligase
CHS: Chalcone synthase
CHI: Chalcone isomerase
F3H: Flavone 3-hydroxylase
FLS: Flavonol synthase
DFR: Dihydroflavonol reductase
UFGT: UDP-flavonoid glucosyl transferase
LDOX: Leucoanthocyanidin dioxygenase
PDS: Phytoene desaturase
ZDS: ζ-carotene desaturase
β-LCY: Lycopene β-cyclase
ε-LCY: Lycopene ε –cyclase
DWARF27: β-carotene isomerase
CCD7: 9-cis-β-carotene 9′,10′-cleaving dioxygenase
CCD8: Carlactone synthase
ZEP: Zeaxanthin epoxidase
LUT1: Epsilon hydroxylase
VDE: Violaxanthin de-epoxidase
NCED: 9-cis-epoxycarotenoid dioxygenase
CYP707A: (+)-abscisic acid 8′-hydroxylase
ABA2: Xanthoxin dehydrogenase
bHLH: Basic helix loop helix
ERF: Ethylene response elements

## References

[1] Ahmed S, Rashid MAR, Zafar SA, Azhar MT, Waqas M, Uzair M (2021). Genome-wide investigation and expression analysis of APETALA-2 transcription factor subfamily reveals its evolution, expansion and regulatory role in abiotic stress responses in Indica Rice (Oryza sativa L. ssp. indica). Genomics.

[2] Ahrazem O, Rubio-Moraga A, Trapero A, Gómez-Gómez L (2011). Developmental and stress regulation of gene expression for a 9-cis-epoxycarotenoid dioxygenase, CstNCED, isolated from Crocus sativus stigmas. J Exp Bot.

[3] Aida R, Ohmiya A, Onozaki T (2018). Current researches in ornamental plant breeding. Breed Sci.

[4] Alquézar B, Zacarías L, Rodrigo MJ (2009). Molecular and functional characterization of a novel chromoplast-specific lycopene beta-cyclase from citrus and its relation to lycopene accumulation. J Exp Bot.

[5] An JP, Zhang XW, Bi SQ, You CX, Wang XF, Hao YJ (2020). The ERF transcription factor MdERF38 promotes drought stress-induced anthocyanin biosynthesis in apple. Plant J.

[6] Anders S, Huber W (2010). Differential expression analysis for sequence count data. Genome Biol.

[7] Cazzonelli CI, Pogson BJ (2010). Source to sink: regulation of carotenoid biosynthesis in plants. Trends Plant Sci.

[8] Chase MW, Cameron KM, Freudenstein JV, Pridgeon AM, Salazar G, van den Berg C (2015). An updated classification of Orchidaceae. Bot J Linn Soc.

[9] Chen C, Chen C (2015). Overview of plant pigments. Pigments In fruits and vegetables: genomics and dietetics.

[10] Chiou C-Y, Pan H-A, Chuang Y-N, Yeh K-W (2010). Differential expression of carotenoid-related genes determines diversified carotenoid coloration in floral tissues of Oncidium cultivars. Planta.

[11] Chiou C-Y, Yeh K-W (2008). Differential expression of MYB gene (OgMYB1) determines color patterning in floral tissue of Oncidium Gower Ramsey. Plant Mol Biol.

[12] Clotault J, Peltier D, Berruyer R, Thomas M, Briard M, Geoffriau E (2008). Expression of carotenoid biosynthesis genes during carrot root development. J Exp Bot.

[13] Cui X, Deng J, Huang C, Tang X, Li X, Li X, et al. Transcriptomic analysis of the anthocyanin biosynthetic pathway reveals the molecular mechanism associated with purple color formation in Dendrobium Nestor. Life (Basel). 2021;11. 10.3390/life11020113 PMC7912934 33540822

[14] Dasgupta K, Thilmony R, Stover E, Oliveira ML, Thomson J (2017). Novel R2R3-MYB transcription factors from Prunus americana regulate differential patterns of anthocyanin accumulation in tobacco and citrus. GM Crops Food.

[15] Davies K, Schwinn K. Molecular biology and biotechnology of flower pigments. In: Plant developmental biology-biotechnological perspectives: Springer; 2010. p. 161–87.

[16] Fang J, Guo T, Xie Z, Chun Y, Zhao J, Peng L (2021). The URL1-ROC5-TPL2 transcriptional repressor complex represses the ACL1 gene to modulate leaf rolling in rice. Plant Physiol.

[17] Gonzalez A, Zhao M, Leavitt JM, Lloyd AM (2008). Regulation of the anthocyanin biosynthetic pathway by the TTG1/bHLH/Myb transcriptional complex in Arabidopsis seedlings. Plant J.

[18] González-Guzmán M, Apostolova N, Bellés JM, Barrero JM, Piqueras P, Ponce MR (2002). The short-chain alcohol dehydrogenase ABA2 catalyzes the conversion of xanthoxin to abscisic aldehyde. Plant Cell.

[19] Grabherr MG, Haas BJ, Yassour M, Levin JZ, Thompson DA, Amit I (2011). Full-length transcriptome assembly from RNA-Seq data without a reference genome. Nat Biotechnol.

[20] Grotewold E (2006). The genetics and biochemistry of floral pigments. Annu Rev Plant Biol.

[21] Guo N, Han S, Zong M, Wang G, Zheng S, Liu F (2019). Identification and differential expression analysis of anthocyanin biosynthetic genes in leaf color variants of ornamental kale. BMC Genomics.

[22] He R, Zhuang Y, Cai Y, Agüero CB, Liu S, Wu J (2018). Overexpression of 9-cis-Epoxycarotenoid Dioxygenase Cisgene in grapevine increases drought tolerance and results in pleiotropic effects. Front Plant Sci.

[23] Hichri I, Barrieu F, Bogs J, Kappel C, Delrot S, Lauvergeat V (2011). Recent advances in the transcriptional regulation of the flavonoid biosynthetic pathway. J Exp Bot.

[24] Holton TA, Cornish EC (1995). Genetics and biochemistry of anthocyanin biosynthesis. Plant Cell.

[25] Hsu CC, Chen YY, Tsai WC, Chen WH, Chen HH (2015). Three R2R3-MYB transcription factors regulate distinct floral pigmentation patterning in Phalaenopsis spp. Plant Physiol.

[26] Huang B, Rong H, Ye Y, Ni Z, Xu M, Zhang W (2020). Transcriptomic analysis of flower color variation in the ornamental crabapple (Malus spp.) half-sib family through Illumina and PacBio sequel sequencing. Plant Physiol Biochem.

[27] Jaakola L, Määttä K, Pirttilä AM, Törrönen R, Kärenlampi S, Hohtola A (2002). Expression of genes involved in anthocyanin biosynthesis in relation to anthocyanin, proanthocyanidin, and flavonol levels during bilberry fruit development. Plant Physiol.

[28] Jaakola L, Poole M, Jones MO, Kämäräinen-Karppinen T, Koskimäki JJ, Hohtola A (2010). A SQUAMOSA MADS box gene involved in the regulation of anthocyanin accumulation in bilberry fruits. Plant Physiol.

[29] Jia K-P, Baz L, Al-Babili S (2017). From carotenoids to strigolactones. J Exp Bot.

[30] Kim DH, Park S, Lee JY, Ha SH, Lim SH. Enhancing flower color through simultaneous expression of the B-Peru and mPAP1 transcription factors under control of a flower-specific promoter. Int J Mol Sci. 2018;19. 10.3390/ijms19010309 PMC5796253 29361688

[31] Krieger E, De Keyser E, De Riek J. Do new breeding techniques in ornamentals and fruits Lead to essentially derived varieties? Front Plant Sci. 2020;10. 10.3389/fpls.2019.01612 PMC7064615 32194575

[32] Landi M, Tattini M, Gould KS (2015). Multiple functional roles of anthocyanins in plant-environment interactions. Environ Exp Bot.

[33] Lee YI, Chang FC, Chung MC (2011). Chromosome pairing affinities in interspecific hybrids reflect phylogenetic distances among lady's slipper orchids (Paphiopedilum). Ann Bot.

[34] Li B, Xia Y, Wang Y, Qin G, Tian S (2017). Characterization of genes encoding key enzymes involved in anthocyanin metabolism of kiwifruit during storage period. Front Plant Sci.

[35] Li C, Qiu J, Ding L, Huang M, Huang S, Yang G (2017). Anthocyanin biosynthesis regulation of DhMYB2 and DhbHLH1 in Dendrobium hybrids petals. Plant Physiol Biochem.

[36] Li H, Qiu J, Chen F, Lv X, Fu C, Zhao D (2012). Molecular characterization and expression analysis of dihydroflavonol 4-reductase (DFR) gene in Saussurea medusa. Mol Biol Rep.

[37] Li X, Zhao W, Sun X, Huang H, Kong L, Niu D (2013). Molecular cloning and characterization of violaxanthin de-epoxidase (CsVDE) in cucumber. PLoS One.

[38] Liu C, Yu H, Rao X, Li L, Dixon RA (2021). Abscisic acid regulates secondary cell-wall formation and lignin deposition in <em>Arabidopsis thaliana</em> through phosphorylation of NST1. Proc Natl Acad Sci.

[39] Livak KJ, Schmittgen TD (2001). Analysis of relative gene expression data using real-time quantitative PCR and the 2− ΔΔCT method. Methods.

[40] Maoka T (2020). Carotenoids as natural functional pigments. J Nat Med.

[41] Marciano DC, Lua RC, Katsonis P, Amin SR, Herman C, Lichtarge O (2014). Negative feedback in genetic circuits confers evolutionary resilience and capacitance. Cell Rep.

[42] Meng Y, Wang Z, Wang Y, Wang C, Zhu B, Liu H (2019). The MYB activator WHITE PETAL1 associates with MtTT8 and MtWD40-1 to regulate carotenoid-derived flower pigmentation in Medicago truncatula. Plant Cell.

[43] Nishihara M, Nakatsuka T (2011). Genetic engineering of flavonoid pigments to modify flower color in floricultural plants. Biotechnol Lett.

[44] Park H, Kreunen SS, Cuttriss AJ, DellaPenna D, Pogson BJ (2002). Identification of the carotenoid isomerase provides insight into carotenoid biosynthesis, prolamellar body formation, and photomorphogenesis. Plant Cell.

[45] Patil S, Zafar SA, Uzair M, Zhao J, Fang J, Li X (2019). An improved Mesocotyl elongation assay for the rapid identification and characterization of Strigolactone-related Rice mutants. Agronomy.

[46] Qin X, Zeevaart JA (1999). The 9-cis-epoxycarotenoid cleavage reaction is the key regulatory step of abscisic acid biosynthesis in water-stressed bean. Proc Natl Acad Sci.

[47] Ramsay NA, Glover BJ (2005). MYB–bHLH–WD40 protein complex and the evolution of cellular diversity. Trends Plant Sci.

[48] Rebecca O, Boyce AN, Chandran S (2010). Pigment identification and antioxidant properties of red dragon fruit (Hylocereus polyrhizus). Afr J Biotechnol.

[49] Sagawa JM, Stanley LE, LaFountain AM, Frank HA, Liu C, Yuan Y-W (2016). An R2R3-MYB transcription factor regulates carotenoid pigmentation in Mimulus lewisii flowers. New Phytol.

[50] Sainz MB, Grotewold E, Chandler VL (1997). Evidence for direct activation of an anthocyanin promoter by the maize C1 protein and comparison of DNA binding by related Myb domain proteins. Plant Cell.

[51] Sheng L, Xia W, Zang S, Zeng Y, Yuan X, Ning G (2018). Transcriptome-sequencing analyses reveal putative genes related to flower color variation in Chinese Rosa rugosa. Acta Physiol Plant.

[52] Snowden KC, Simkin AJ, Janssen BJ, Templeton KR, Loucas HM, Simons JL (2005). The decreased apical dominance1/Petunia hybrida CAROTENOID CLEAVAGE DIOXYGENASE8 gene affects branch production and plays a role in leaf senescence, root growth, and flower development. Plant Cell.

[53] Stanley L, Yuan YW (2019). Transcriptional regulation of carotenoid biosynthesis in plants: so many regulators, so little consensus. Front Plant Sci.

[54] Takahashi R, Yamagishi N, Yoshikawa N (2013). A MYB transcription factor controls flower color in soybean. J Hered.

[55] Tanaka Y, Sasaki N, Ohmiya A (2008). Biosynthesis of plant pigments: anthocyanins, betalains and carotenoids. Plant J.

[56] Tian J, Chen M-c, Zhang J, Li K-t, Song T-t, Zhang X (2017). Characteristics of dihydroflavonol 4-reductase gene promoters from different leaf colored Malus crabapple cultivars. Horticult Res.

[57] Tian J, Peng Z, Zhang J, Song T, Wan H, Zhang M (2015). McMYB10 regulates coloration via activating McF3′H and later structural genes in ever-red leaf crabapple. Plant Biotechnol J.

[58] Trapnell C, Williams BA, Pertea G, Mortazavi A, Kwan G, Van Baren MJ (2010). Transcript assembly and quantification by RNA-Seq reveals unannotated transcripts and isoform switching during cell differentiation. Nat Biotechnol.

[59] van der Krol AR, Mur LA, de Lange P, Mol JNM, Stuitje AR (1990). Inhibition of flower pigmentation by antisense CHS genes: promoter and minimal sequence requirements for the antisense effect. Plant Mol Biol.

[60] Vogel C, Marcotte EM (2012). Insights into the regulation of protein abundance from proteomic and transcriptomic analyses. Nat Rev Genet.

[61] Vu H-T, Vu Q-L, Nguyen T-D, Tran N, Nguyen T-C, Luu P-N (2020). Genetic diversity and identification of Vietnamese Paphiopedilum species using DNA sequences. Biology.

[62] Wang F, Chen L, Chen H, Chen S, Liu Y. Analysis of flavonoid metabolites in citrus peels (Citrus reticulata "Dahongpao") using UPLC-ESI-MS/MS. Molecules. 2019;24. 10.3390/molecules24152680 PMC6696472 31344795

[63] Waqas MA, Wang X, Zafar SA, Noor MA, Hussain HA, Azher Nawaz M, et al. Thermal stresses in maize: effects and management strategies. Plants (Basel). 2021;10. 10.3390/plants10020293 PMC7913793 33557079

[64] Wu X-M, Lim S-H, Yang W-C (2003). Characterization, expression and phylogenetic study of R2R3-MYB genes in orchid. Plant Mol Biol.

[65] Xie Y, Tan H, Ma Z, Huang J (2016). DELLA proteins promote anthocyanin biosynthesis via sequestering MYBL2 and JAZ suppressors of the MYB/bHLH/WD40 complex in Arabidopsis thaliana. Mol Plant.

[66] Yahyaa M, Ali S, Davidovich-Rikanati R, Ibdah M, Shachtier A, Eyal Y (2017). Characterization of three chalcone synthase-like genes from apple (Malus x domestica Borkh.). Phytochemistry.

[67] Yang X, Xia X, Zhang Z, Nong B, Zeng Y, Wu Y (2019). Identification of anthocyanin biosynthesis genes in rice pericarp using PCAMP. Plant Biotechnol J.

[68] Zafar SA, Hameed A, Ashraf M, Khan AS, Qamar ZU, Li X (2020). Agronomic, physiological and molecular characterisation of rice mutants revealed the key role of reactive oxygen species and catalase in high-temperature stress tolerance. Funct Plant Biol.

[69] Zafar SA, Uzair M, Khan MR, Patil SB, Fang J, Zhao J (2021). DPS1 regulates cuticle development and leaf senescence in rice. Food Energy Secu.

[70] Zafar SA, Zaidi SS, Gaba Y, Singla-Pareek SL, Dhankher OP, Li X (2020). Engineering abiotic stress tolerance via CRISPR/ Cas-mediated genome editing. J Exp Bot.

[71] Zhang S, Zhang A, Wu X, Zhu Z, Yang Z, Zhu Y (2019). Transcriptome analysis revealed expression of genes related to anthocyanin biosynthesis in eggplant (Solanum melongena L.) under high-temperature stress. BMC Plant Biol.

[72] Zhao A, Cui Z, Li T, Pei H, Sheng Y, Li X, et al. mRNA and miRNA expression analysis reveal the regulation for flower spot patterning in Phalaenopsis 'Panda'. Int J Mol Sci. 2019;20. 10.3390/ijms20174250 PMC6747512 31480267

[73] Zhao D, Tao J (2015). Recent advances on the development and regulation of flower color in ornamental plants. Front Plant Sci.

[74] Zhao J, Zhao L, Zhang M, Zafar SA, Fang J, Li M, et al. Arabidopsis E3 ubiquitin ligases PUB22 and PUB23 negatively regulate drought tolerance by targeting ABA receptor PYL9 for degradation. Int J Mol Sci. 2017;18. 10.3390/ijms18091841 PMC5618490 28837065

