# Supplementary material for: Comparative transcriptome analysis identified important genes and regulatory pathways for flower color variation in Paphiopedilum hirsutissimum
Source: BMC Plant Biol. 2021 Oct 27;21:495. doi: 10.1186/s12870-021-03256-3 (PMC8549352; doi:10.1186/s12870-021-03256-3)
Supplement: Supplementary file 1 — Additional file 1: Table S1: Primer sequences of genes used for qRT-PCR and vector construction. [file 12870_2021_3256_MOESM1_ESM.docx]

**Primers for vector construction and qRT-PCR**

| MYB61-2300-F | AGAACACGGGGGACGAGCTCATGGGGAGGCACTCTTGCTGC |  |
| --- | --- | --- |
| MYB61-2300-R | ACCATGGTGTCGACTCTAGAACATGCTATGTCTCTCTGGCACTC |  |
| ERF087-2300-F | AGAACACGGGGGACGAGCTCCCAACGCAAGACCTATCCAGC |  |
| ERF087-2300-R | ACCATGGTGTCGACTCTAGAGAAGAAATAGGTGGATGAAGAAGAGC |  |
| DN26827-2300-F | AGAACACGGGGGACGAGCTCCAACAATCTTCTATGGCAGACATCC |  |
| DN26827-2300-R | ACCATGGTGTCGACTCTAGACGACGCCGGGGCAGG |  |
| DN49122-2300-F | AGAACACGGGGGACGAGCTCATGGTTCGGGGAAAGACGGAGA |  |
| DN49122-2300-R | ACCATGGTGTCGACTCTAGAATATAAGGTTCCTCTCAACAAATGGGC |  |
| DN57904-2300-F | AGAACACGGGGGACGAGCTCATGGAGGAGCTCATCTCGCATTC |  |
| DN57904-2300-R | ACCATGGTGTCGACTCTAGATGTGCGGCCGCTCGCAG |  |
| DN40678-Actin-F | gCACTACTCCAAggAACCCC |  |
| DN40678-Actin-R | gACgCCAAgggTgATCTTgA |  |
| DN100683-F | TCTgCCgACCAAACAAAgA |  |
| DN100683-R | ggTggATTgAAgCCggATAA |  |
| DN102311-F | TggTTgggCATCTCTCTTTC |  |
| DN102311-R | CTgCTgAggTgTgCATAAgA |  |
| DN102917-F | CgAAgTggAAgTAgAggTgATTT |  |
| DN102917-R | TCgATgCTCTTgTACTgATTgg |  |
| DN103576-F | AgCggAggATTgCTTCTTATC |  |
| DN103576-R | TTgCTCATTggCCACTACTT |  |
| DN104112-F | CAgAACCATCgAgggAgTTT |  |
| DN104112-R | CCTgTgTCgCTTCCTgTATT |  |
| DN57122-F | CTCCACCgAATCCTTCCAAA |  |
| DN57122-R | CACTCCTgCACAACCACTAT |  |
| DN59880-F | CCAgATCgCTTgAgATgAgAAg |  |
| DN59880-R | TTCgACgAACAgCATggATAA |  |
| DN64728-F | gCCCTTCACAACCgTTATCT |  |
| DN64728-R | CTATgTgATgCTgggTgATgT |  |
| DN64821-F | gAgAAgAgggTAggAggAAAgA |  |
| DN64821-R | CAgTAgCCTAATCAgCTCCAAA |  |
| DN60170-F | gATCgTCTgCTTCAggTACg |  |
| DN60170-R | gAgATgAgATCggTAgggAAATg |  |
