# Supplementary figures and images for: Comparative transcriptome analysis identified important genes and regulatory pathways for flower color variation in Paphiopedilum hirsutissimum
Source: BMC Plant Biol. 2021 Oct 27;21:495. doi: 10.1186/s12870-021-03256-3 (PMC8549352; doi:10.1186/s12870-021-03256-3)

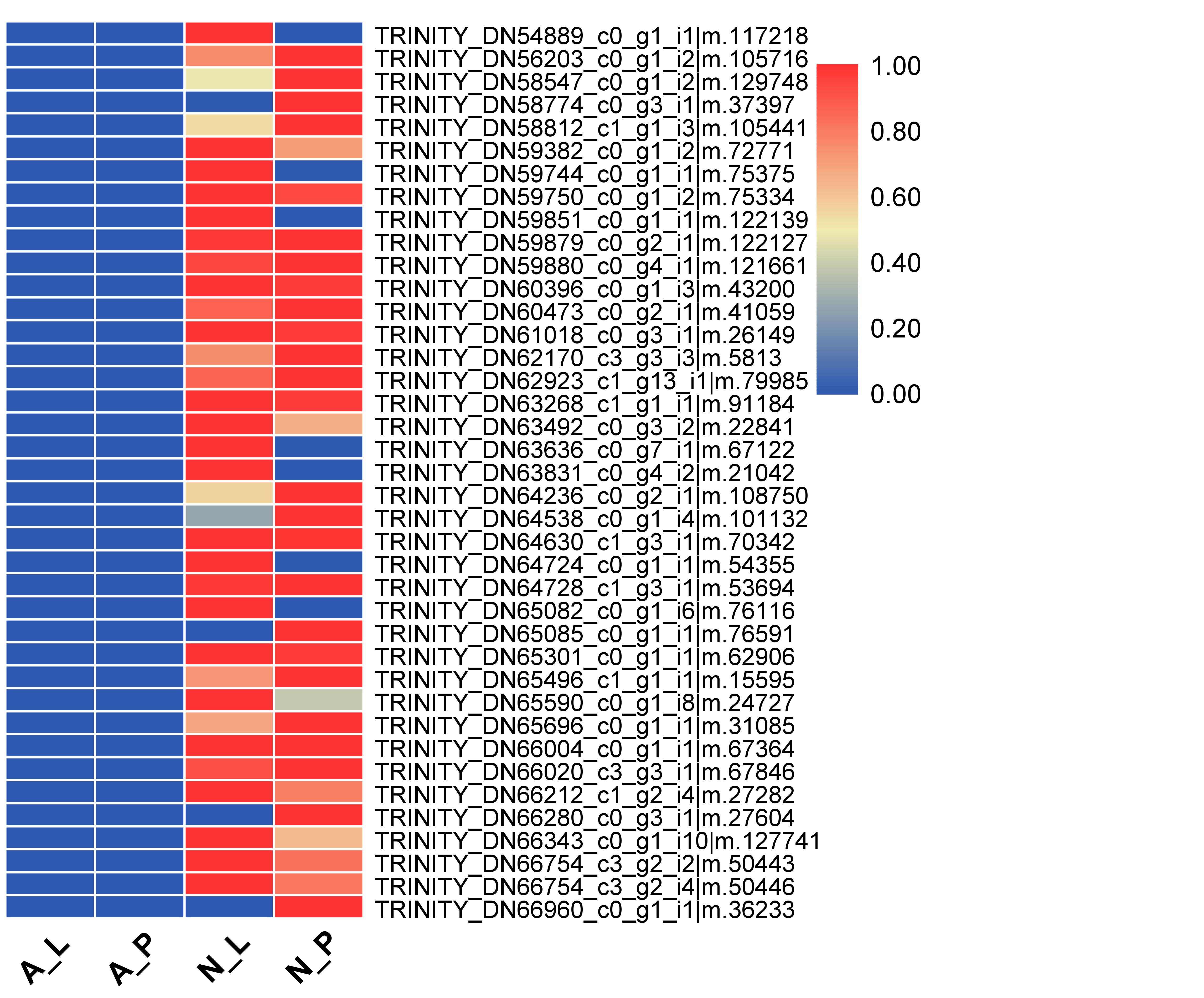

Supplement: Supplementary file 4 — Additional file 4: Figure S1a: DEGs expressed only in normal flower tissues. [file 12870_2021_3256_MOESM4_ESM.jpg]

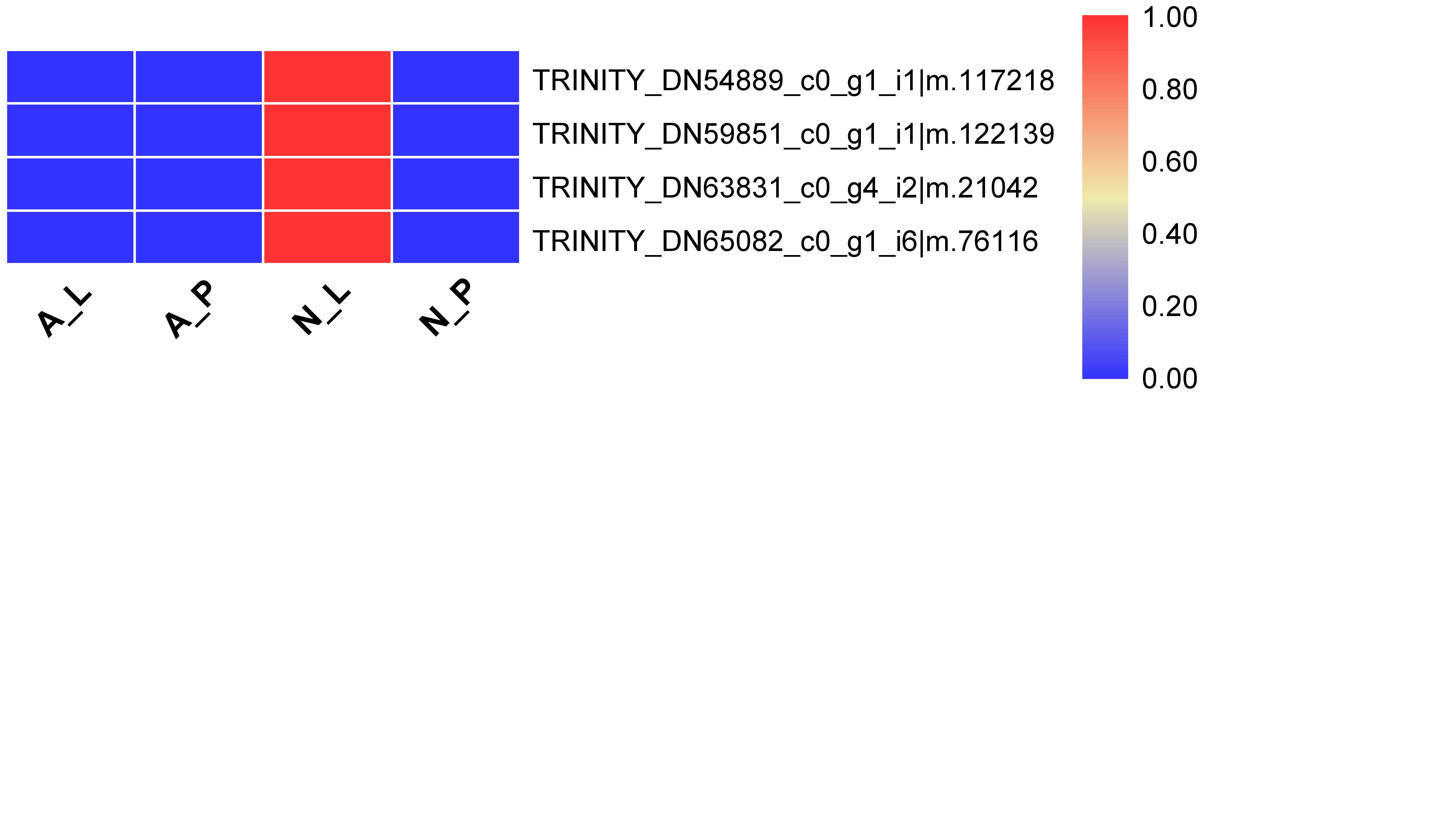

Supplement: Supplementary file 5 — Additional file 5: Figure S1b: DEGs expressed only in normal labellum. [file 12870_2021_3256_MOESM5_ESM.jpg]

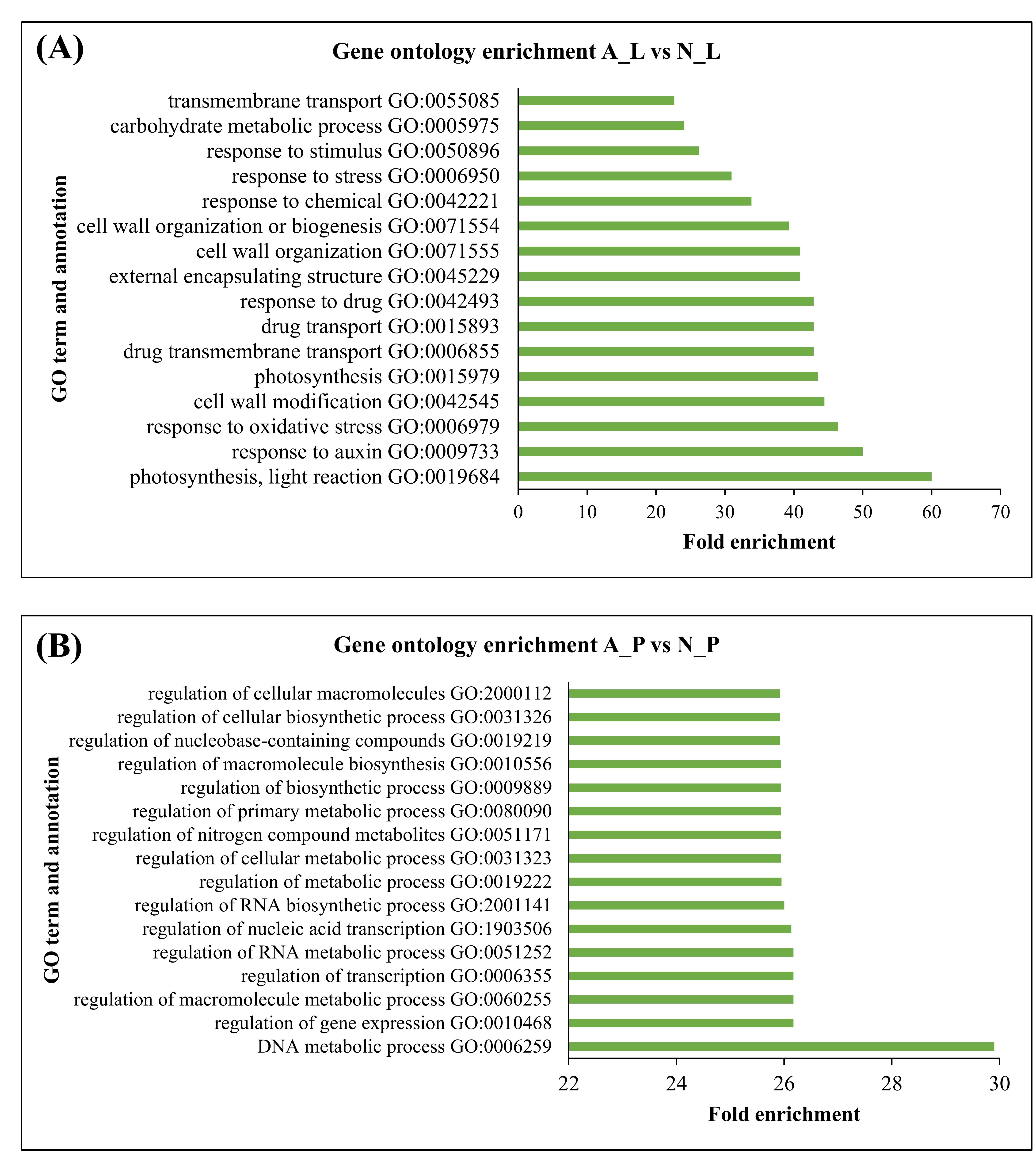

Supplement: Supplementary file 6 — Additional file 6: Figure S2: Gene ontology enrichment analysis among normal and albino tissues. [file 12870_2021_3256_MOESM6_ESM.jpg]

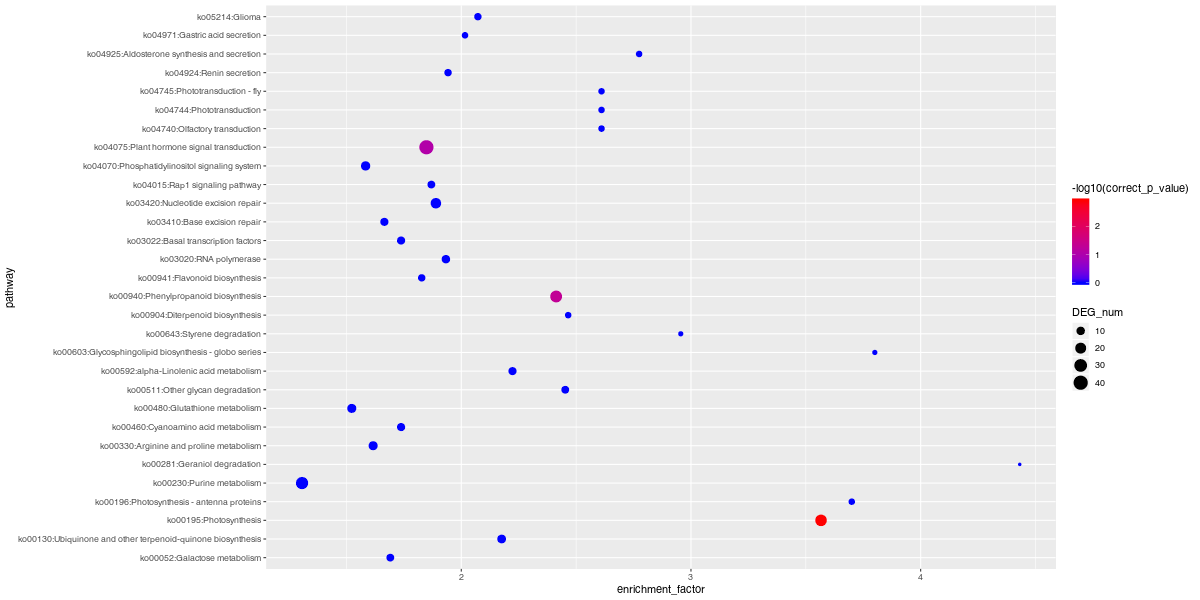

Supplement: Supplementary file 7 — Additional file 7: Figure S3A: KEGG enrichment analysis among normal and albino labellum. [file 12870_2021_3256_MOESM7_ESM.png]

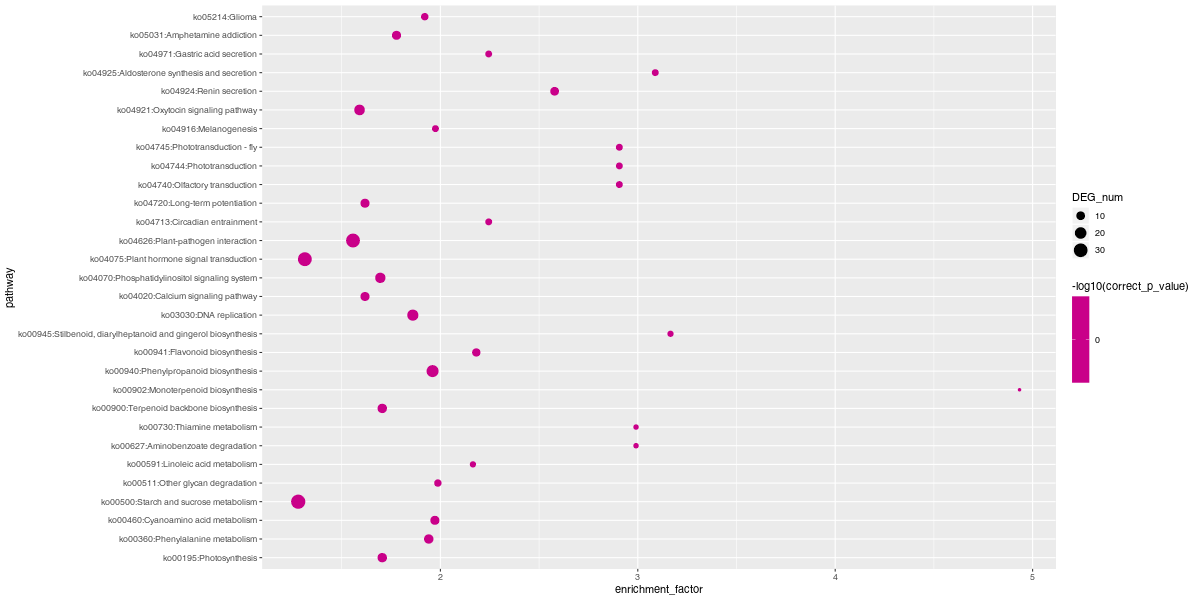

Supplement: Supplementary file 8 — Additional file 8: Figure S3B: KEGG enrichment analysis among normal and albino petals. [file 12870_2021_3256_MOESM8_ESM.png]
